# Characterization of food color additives and evaluation of their acute toxicity in Wistar albino rats

**DOI:** 10.14202/vetworld.2024.2329-2337

**Published:** 2024-10-17

**Authors:** D. A. Qasim, I. J. Lafta

**Affiliations:** 1 Market Research and Consumer Protection Center, University of Baghdad, Baghdad, Iraq; 2 Department of Microbiology, College of Veterinary Medicine, University of Baghdad, Baghdad, Iraq

**Keywords:** acute toxicity, food colors, Fourier-transform infrared spectroscopy, lethal dose 50, range-finding study

## Abstract

**Background and Aim:**

The use of food dyes can cause certain diseases, such as anemia and indigestion, along with other disorders, tumors, and even cancer. Therefore, this study aimed to determine the chemical nature and toxicity of some commercial dyes locally used in processed foods compared with standard food dyes.

**Materials and Methods:**

Three types of standard and commercial food color additives (Sunset Yellow, Tartrazine, and Carmoisine) were extensively examined. The chemical structures and functional groups of the dyes were evaluated by Fourier-transform infrared (FTIR) spectroscopy. The melting temperatures of the dyes were also determined by chemical thermal analysis. The acute toxicity test to evaluate the standard and commercial food color safety was estimated by a range-finding study using 150 Wistar albino rats. Sub-groups were administered one of the three colors under study at doses of 2, 3, 4, and 5 g/kg body weight (BW) orally for 7 days. When no mortality was observed, an additional 15 g/kg BW was administered. Concerning the median lethal dose 50 (LD_50_), 38 rats were exploited using the up-and-down method.

**Results:**

Commercial dyes had lower melting points than standard colors. Regarding the range-finding study, rats receiving different doses of the dyes exhibited no signs of toxicity, no deaths, and no clinical or gross pathological signs throughout the 7 days of the experiment. However, the animals that were dosed with 15 g/kg BW of each dye showed signs of loss of appetite, tachycardia, drowsiness, and eventual death. The LD_50_ values of the commercial food dyes, particularly Sunset Yellow and Carmoisine, were lower than those of the standard dyes.

**Conclusion:**

Commercial food colors were more toxic to rats than standard food colors. Differences were observed between the purity of the standard and commercial dyes, and the latter ones contained different percentages of salt, indicating the occurrence of fraud in commercial markets.

## Introduction

According to the Food and Drug Administration definition, any substance added to food or non-food applications, such as colorings, flavorings, and preservatives, is considered an additive [1]. Natural food dyes were known before the middle of the nineteenth century, when materials with colorful properties were mainly extracted from animal or plant sources. Subsequently, at the beginning of the 20^th^ century, natural dyes were almost completely replaced by synthetic dyes [2]. Due to the properties of synthetic dyes, including their homogeneity, low cost, and stability, in addition to their availability in many different colors, synthetic colors have been widely used because these properties increase their quality and esthetics [3]. Food color additives have three distinctive types (anionic, non-anionic, or cationic). According to the chemical structure and composition of the chromophores, different groups of dyes are present, ranging from 20 to 30 types. The most important groups of dyes are anionic and non-anionic dyes, which contain azo-chromophores or anthraquinone [4]. Dyes containing the azo-chromophore group have been used in many applications, including commercial food production and non-food applications, such as manufacturing industries, cosmetics, medicines, home design artifacts, and medical devices [5]. The azo-chromophore (-N=N-) group linked to the aromatic rings constitutes more than 65% of the commonly used commercialized dyes due to their stability in foods, high solubility, and low cost [6].

Most of the dyes containing azo groups can cause allergic reactions and severe eye irritation, adding to high toxicity if they are absorbed through skin contact, inhaled, or consumed for a prolonged period, and can lead to the risk of cancer in 60%–70% of cases [7]. In addition to their interference with the photosynthesis processes of plants and marine organisms, phytopathogens can lead to long-term hazards due to resistance to treatment with standard techniques [8]. Furthermore, if fish and other living organisms ingest these dyes, they will turn into toxic intermediates due to their ability to metabolize these dyes, leading to a negative impact on the health of fish and predatory animals, resulting in their poisoning and death [9]. Sunset Yellow (Yellow 6, SY or E110), Carmoisine (CAR, azorubine or E122), and Tartrazine (Yellow 5 or E102) are among the most common azo food dyes used in food manufacturing [10]. Sunset Yellow is an azo orange dye derived from petroleum. It is polar, has poor solubility in ethanol, dissolves well in water, and its aqueous solutions are yellow-orange in color and turn brownish in neutral and alkaline solutions [11]. It is a mono-azo di-sulfonated hydroxyl dye, its chemical name is disodium 2-hydroxy-1-(4-sulphonatophenylazo) naphthalene-6-sulphonate (C_16_H_10_N_2_Na_2_O_7_S_2_), with 452.37 g/mol molecular weight [12]. It is usually available in various food products, for example, sweets, jams, aromatic and fermented beverages, desserts, chewing gum, ice cream, spices, soups, and jellies. It is also found inside living organisms, such as fish roe and crustaceans, to give them orange-yellow color [12]. Carmoisine is a bright red azo powder that is widely used in the production of foods, cosmetics, and pharmaceutical products [13]. The chemical name of Carmoisine is di-sodium 4-hydroxy3-(4-sulphonato-1-naphthylazo) naphthalene-1-sulphonate (C_20_H_12_N_2_Na_2_O_7_S_2_), which has a molar mass of 502.44 g/mol [13]. Carmoisine is a biologically active substance, but its absorption is limited in the digestive system. However, free sulfonated aromatic amines are highly absorbed and reach the systemic circulation [13]. The purity percentage of Carmoisine used for commercial purposes and experiments must exceed 85%, and the remaining 15% belongs to sodium salts, either sodium sulfate or sodium chloride salts [14]. Tartrazine is an azo dye that gives a bright lemon-yellow color, and its solubility in water is well known; however, in ethanol, it is poorly soluble [15]. It is a synthetic food color produced from coal tar and has been widely used in food production and pharmaceutical and cosmetic companies [12, 15]. Being one of the most widely used dyes, as with many other synthetic compounds, it has side effects on the human body. It can cause perturbation, anemia, leucopenia, renal and hepatic toxicity, and other symptoms and diseases in rats [16]. The emergence of symptoms and disorders such as allergic reactions and attention deficit disorders in humans, especially children, has also been demonstrated [17]. The degree of toxicity of food dyes depends on the chemical interactions between the food color and other ingredients. The percentage of toxicity may be affected by the degree of absorption, metabolism, and excretion of the food product containing the dyes, and the toxicity has been linked to oxidative stress caused by the generated superoxide, reactive hydroxyl group, aryl amine, and aromatic amine [18]. There are many diseases, such as indigestion and anemia, that can be caused by prolonged use of food dyes, leading to damage of the kidneys, spleen, liver, and possibly the brain, causing problems with growth and vision, ending with blindness, allergic reactions, and cancer [19].

Specifically, understanding the risks of commercial versus standard dyes for mostly used food color additives, such as Sunset Yellow, Tartrazine, and Carmoisine, could be an area of interest. Therefore, this study aimed to determine whether the daily intake of the most common food colors at different oral concentrations was safe or not, using the range-finding study and median lethal dose 50 (LD_50_) estimation in rats, as well as characterization of the chemical nature of local commercial dyes compared with standard imported colors.

## Materials and Methods

### Ethical approval

This study was approved by the Research Ethics Committee, College of Veterinary Medicine, University of Baghdad, Baghdad, Iraq, according to the Animal Utilization Protocol Certification, number 2282/PG on December 6, 2023

### Study period and location

The study was performed from January 2023 to March 2024 in the College of Veterinary Medicine, University of Baghdad, Baghdad, Iraq.

### Food color additives

This study examined food color additives, including Sunset Yellow, Tartrazine, and Carmoisine (Roha Dyechem, India). The commercial dyes used locally in processed foods were bought from the local market, Al-Shorja, Baghdad, Iraq, and compared with standard colors produced by Sigma-Aldrich (Germany).

### Experimental animals

In total, 188 Wistar albino rats of both sexes with a body weight ranging from 90 to 160 g and aged 6–12 weeks were maintained in the animal house of the College of Veterinary Medicine, University of Baghdad. They were maintained in an air-conditioned room (25°C ± 1°C) with 12 h light: 12 h dark cycle. The rats were fed a commercially available standard diet and had unlimited access to food and water during the experiment. The animals were left to be acclimatized for at least 2 weeks before starting the experiment.

### Determination of the food color melting point

A closed-end melting point capillary tube (Marienfeld, Germany) was filled to one-third with each dye as a powder and then placed in an electro-thermal melting point apparatus (Gallenkamp, UK). The temperature at which the dye sample became liquid was carefully observed and recorded.

### Fourier-transform infrared (FTIR) spectroscopy

FTIR spectroscopy (Shimadzu IR-Affinity, Japan) was used in the present study to identify functional groups and detect the purity of food colors in their commercial and standard forms. The FTIR spectra were scanned in the range 4000–450 cm^-1^ and a resolution of 4 cm^-1^ by spreading food color on a glass slide [20]. The FTIR data were analyzed at the College of Science, University of Baghdad, Baghdad, Iraq.

### Safety assessment of food color additives using a range-finding study

A range-finding experiment was carried out to evaluate the safety of commercial and standard food colors (Sunset Yellow, Tartrazine, and Carmoisine). For this experiment, 150 Wistar albino rats were divided into two groups (each consisting of 75 rats) for standard and commercial food colors. Each group was then organized into five subgroups, each with 15 animals. On the next day, following an overnight fast, 4 different doses (2 g, 3 g, 4 g, and 5 g/kg body weight [BW]) of each food dye were administered. These dyes were prepared by dissolving 2, 3, 4, and 5 g of each food color powder in 20 mL of distilled water. Each rat received 2 mL of the color solution per 100 g BW daily for 7 consecutive days using a gastric gavage needle (18 G). The criteria for determining food color safety were life and death during treatment. When no mortality was observed after 7 days, an additional five rats (in the 5^th^ sub-group) were given a higher dose of 15 g/kg BW of each color using the protocol mentioned above. Throughout the experiment, the animals’ BW, clinical signs, signs of toxicity, abnormal physical and behavioral changes, morbidity, and mortality were closely monitored [21].

### Determining acute toxicity by estimating LD_50_ using the up-and-down method

The up-and-down method of Dixon [22] was adopted to determine the LD_50_ of standard and commercial food dyes. For this experiment, 38 Wistar albino rats were allocated to the Tartrazine, Sunset Yellow, and Carmoisine evaluations. Initially, three rats were used, and they orally received a single dose of each dye using a gastric gavage needle and were monitored for 24 h for the occurrence of death or survival of the animal. The dose given to the next animals was adjusted by either increasing or decreasing the dose to a factor of 20% of the original dose. Depending on the outcome observed in the previous animals, doses were continued to be administered to other animals for three shots until reaching an LD_50_. The LD_50_ values were calculated according to the outcome of death or survival of the rats using the following equation:

LD_50_ = xf + kd

xf = Last dose used in the experiment

d = Difference between doses

k = Factor of change

## Results

### Melting point and FTIR spectroscopy

In this study, the melting point of the commercial dyes (Sunset Yellow, Tartrazine, and Carmoisine) used locally for processing foods was lower than that of the standard form, as illustrated in Table-1.

**Table-1:** Melting points of commercial food colors compared with the standard dyes.

Food color	Commercial dyes (°C)	Standard dyes (°C)
Sunset Yellow	279–282	298–302
Tartrazine	215–219	239–241
Carmoisine	263–266	286–289

**Figure-1:**
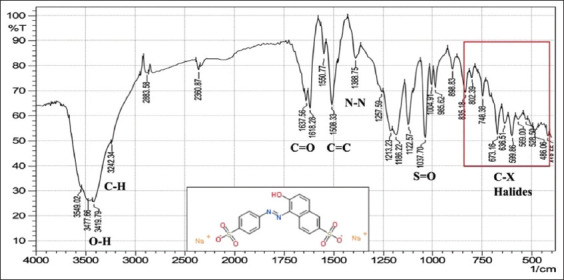
Fourier-transform infrared spectra of the commercial Sunset Yellow powder at wave numbers of 4000–450 cm^-1^.

**Figure-2:**
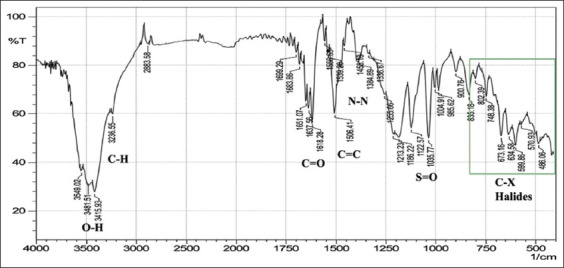
Fourier-transform infrared spectra of standard Sunset Yellow powder at wavenumbers of 4000–450 cm^-1^.

**Figure-3:**
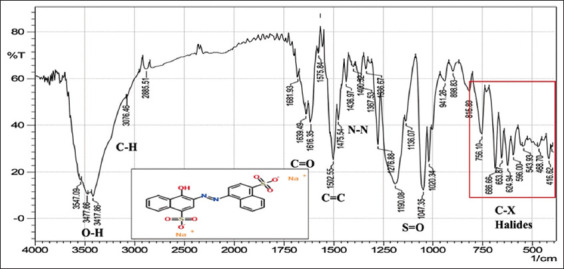
Fourier-transform infrared spectra of the commercial carmoisine powder at wave numbers of 4000–450 cm^-1^.

**Figure-4:**
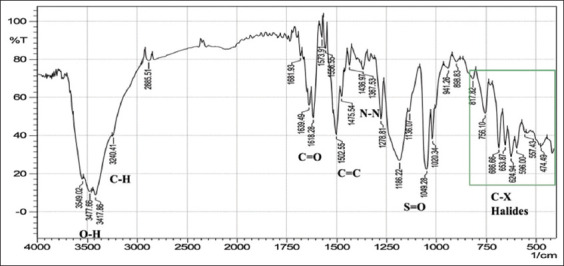
Fourier-transform infrared spectra of the standard carmoisine powder at wave numbers of 4000–450 cm^-1^.

**Figure-5:**
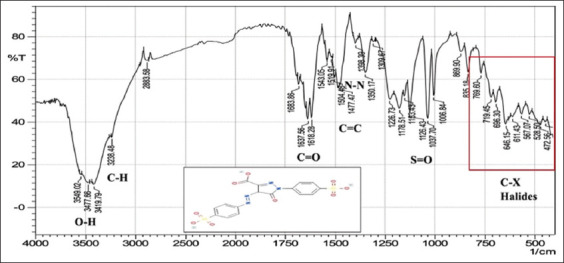
Fourier-transform infrared spectra of the commercial tartrazine powder at wave numbers of 4000–450 cm^-1^.

**Figure-6:**
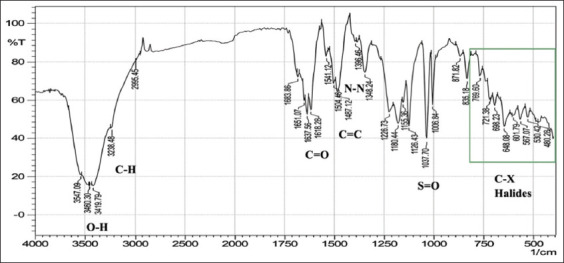
Fourier-transform infrared spectra of standard tartrazine powder at wave numbers of 4000–450 cm^-1^.

With regard to FTIR spectroscopy, analysis of the azo compounds showed a range of wave numbers 4000–450 cm^−1^ associated with the transmittance mode. The FTIR spectrum associated with the C=C adsorbent indicates the presence of peaks relative to various functional groups, and the absorbance peaks at various wavenumbers in the spectrum correspond to individual functional groups. These functional groups were compared for the best matches with libraries of spectra cataloged for the available material FTIR spectrophotometers to confirm the presence of functional groups in the commercial dyes compared with those found in the standard form from Sigma-Aldrich (Germany). The stretching vibration bands of the azo group are shown in Figures-1-6, which present a relationship between the wave numbers of the highest absorbance and the mode of absorption by identifying the bonds that determine the absorbance and the spectroscopy of the food colors. Similar functional groups were observed in both types of dyes (standard and commercial), with minor differences in shape, position, and intensity.

Despite many similar functional groups in the commercial and standard dyes, differences were observed within the C-X group.

### Safety assessment of commercial and standard food colors

The results of the range-finding study showed that the rats in the groups that received 2–5 g/kg BW of each dye had no signs of toxicity, no deaths, no pathological or clinical signs, and no behavioral changes. These observations continued throughout the experiment till the 7^th^ day of dosing (Table-2). Concerning the 5^th^ additional rats that were dosed with relatively higher doses (15 g/kg BW) of each color, the animals revealed signs of loss of appetite, tachycardia, decrease in locomotion, tremor, and then convulsion, which were the distinctive signs observed on the rats before the occurrence of death in the two groups of the commercial and standard food dyes (Table-2).

**Table-2:** Outcomes of a range-finding study performed for commercial and standard food color additives administered to rats.

Dose g/kg BW	Food color	No. of rats given commercial food dyes	No. of dead animals	No. of live animals	No. of rats given standard food dyes	No. of dead animals	No. of live animals
2	Sunset Yellow	5	0	5	5	0	5
	Tartrazine	5	0	5	5	0	5
	Carmoisine	5	0	5	5	0	5
3	Sunset Yellow	5	0	5	5	0	5
	Tartrazine	5	0	5	5	0	5
	Carmoisine	5	0	5	5	0	5
4	Sunset Yellow	5	0	5	5	0	5
	Tartrazine	5	0	5	5	0	5
	Carmoisine	5	0	5	5	0	5
5	Sunset Yellow	5	0	5	5	0	5
	Tartrazine	5	0	5	5	0	5
	Carmoisine	5	0	5	5	0	5
15	Sunset Yellow	5	5	0	5	5	0
	Tartrazine	5	5	0	5	5	0
	Carmoisine	5	5	0	5	5	0

BW=Body weight

### Determining acute toxicity of commercial and standard food color additives by estimating LD_50_ using the up-and-down method in the rat model

In the present study, the LD_50_ of the commercial food color additives Sunset Yellow and Carmoisine were 6.518 g/kg BW and 11.474 g/kg BW (Table-3). The LD_50_ of the standard food dyes was 11.474 g/kg BW. The acute toxicity signs observed in morbid animals after dosing with food colors included depression, anorexia, muscular weakness, weak response to external stimuli, paralysis and inability to move, recumbence, and death. The LD_50_ values of the commercial food dyes were calculated as follows:

**Table-3:** Outcomes of the acute toxicity study (LD_50_) of commercial food color additives administered to rats.

Food color	Last dose (mg/kg BW)	Value of K	Difference between doses (d)	Outcome	LD_50_
Sunset Yellow	8000	–0.741	2000	XXXOXOX	6.518 g/kg BW
Tartrazine	10000	0.737	2000	XXOXOX	11.474 g BW
Carmoisine	8000	–0.741	2000	XXXOXOX	6.518 g/kg BW

X=Death, O=Survival, K=Constant, LD_50_=Lethal dose 50, BW=Body weight

- LD_50_ (Sunset Yellow) = xf + kd= 8+ (−0.741) × 2 = 6.518 g/kg BW orally after 24 h
- LD_50_ (Tartrazine) = xf + kd= 10+ (0.737) × 2 = 11.474 g/kg BW orally after 24 h
- LD_50_ (Carmoisine) = xf + kd= 8+ (−0.741) × 2 = 6.518 g/kg BW orally after 24 h.

The LD_50_ values of the standard food dyes were as follows:

LD_50_ (Sanset Yellow, Tartrazine, and Carmoisine) = xf + kd= 10+ (0.737) × 2 = 11.474 g/kg BW orally after 24 h (Table-4).

**Table-4:** Outcomes of the acute toxicity study (LD_50_) of standard food color additives administered to rats.

Food color	Last dose (mg/kg BW)	Value of K	Difference between doses (d)	Outcome	LD_50_
Sunset Yellow Tartrazine Carmoisine	10000	0.737	2000	XXOXOX	11.474 g BW

X=Death, O=Survival, K=Constant, LD_50_=Lethal dose 50, BW=Body weight

The severity of symptoms was directly proportional to the dose of the food color, and the interval between the onset of toxicity signs and death was lower for the commercial dyes than for the standard ones. Furthermore, the current results of acute toxicity revealed signs of food color toxicity, and the severity of the signs was dosage dependent; that is, when the color dosage increased, more severe toxic signs and symptoms appeared in the animals. Importantly, no morbidity or mortality occurred in any of the groups of animals treated with Tartrazine dye; therefore, this dye can be classified as slightly toxic.

## Discussion

To determine the purity of food color, melting point analysis is crucial for identifying pure substances. This technique involves measuring the temperature at which a solid substance changes from a solid to a liquid state [23]. The decreased melting points of the commercial dyes under investigation can be attributed to the presence of impurities. These changes make commercial dyes require less energy to be converted to the liquid state, leading to a lowering of the melting point or melting of the material over a wide range of temperatures, a phenomenon known as melting point depression. This indicates that commercial dyes may not be suitable for food and non-food applications, especially those requiring high heat treatment stability. These results are close to the findings of Leulescu *et al*. [24], in which the exposure of the Sunset Yellow dye to high temperatures reached 188°C caused the removal of the water molecules present in the dye, accompanied by thermal instability, and the dye began to melt when the temperature reached 330°C. Exposure to temperatures higher than 330°C–406°C caused oxidative decomposition of the dye molecules [24]. It is estimated that 300°C is the highest temperature that can be used to thermally process foods containing a Sunset Yellow. However, temperatures used to prepare foods containing Sunset Yellow should not exceed 188°C–300°C [25]. The melting temperature, decomposition pathways, and thermal effects of Tartrazine were determined by Carabet *et al*. [26]; In that study, Tartrazine was found to be melted in the range of 224.4°C–225.3°C, while at a temperature of 250°C, it started to decompose. Azorubine (Carmoisine) is thermally melted at temperatures as high as 300°C, and it starts to degrade at a maximum temperature of 310°C [27].

FTIR spectroscopy is an efficient technique for characterizing organic compounds. The FTIR analysis of the azo compounds showed a range of wave numbers 4000–450 cm^−1^ associated with the transmittance mode. Similar functional groups were observed in both dyes (standard and commercial). The commercial and standard dyes generally showed peaks similar to those observed by Stuart [28], who stated that they belong to the O-H region, which revealed a broad absorption peak with stretching vibrations ranging from 3500 to 3200 cm^-1^. This confirms the presence of H-bonded hydroxyl groups.

On the other hand, the spectrum exhibiting strongly absorbing peaks at 3000–2850 cm^-1^ indicated C–H stretching frequency, which, in turn, reflects the presence of alkanes. The strong absorption peaks at 1670–1640 cm^−1^, corresponded to the stretching vibration of the carbonyl group (C=O). The C=C bond was observed in weakly absorbing peaks (1600–1475 cm^-1^), and the stretching vibration of the functional alkene group (C=C) indicated the presence of aromatic compounds. Importantly, the typical bands in the studied colors were related to the azo group (–N=N–) stretching as a medium-to-strong band at 1350–1000 cm^-1^ signals for all amines. The asymmetrical stretching vibration of the S=O (SO_3_-H) group appeared at 1050 cm^-1^ with a strong absorption peak. These functional groups might be responsible for IR adsorption and act as bioactive compounds. Despite many similar functional groups in the standard and commercial dyes, differences are present within the C-X (Halogen) group, which includes fluoride, chloride, bromide, and iodide. The appearance of multiple strong absorption peak signals at <667 cm^-1^ to 1400 cm^-1^ in the commercial dyes compared with the peaks of the standard colors indicates the presence of large percentages of salts added to the commercial dyes, resulting in changes in their components and decreasing their purity relative to the standard dyes. Therefore, commercial dyes are considered impure due to an increase in the number of halides.

Range-finding studies are more favorable for estimating the expectations of the drug, supplement, or food ingredient to determine their overall safety [29, 30]. Thus, this investigation was conducted to evaluate the safety of commercial food additive dyes compared with standard food colors. The results of the present study showed that rats that received 2–5 g/kg BW of each dye had no signs of toxicity, no deaths, no pathological or clinical signs, and no behavioral changes. These observations were continued throughout the experiment till the 7^th^ day of dosing. Regarding the 5^th^ additional rats that received 15 g/kg BW of each color, the animals exhibited signs of loss of appetite, tachycardia, decreased locomotion, tremor, and then convulsion. These results agree with those of the study of Park *et al*. [31], in which the researchers conducted a 7-day dose range study (0, 250, 500, 1000, 2000, and 5000 mg/kg BW) for the red-coloring additive (Carmoisine), and their findings showed neither mortality nor abnormal clinical signs. Several short-term studies on the toxicity of Tartrazine dye were performed in dogs, cats, and rats by Gao *et al*. [32] and Rychen *et al*. [33]. The results of these studies did not demonstrate any adverse effects associated with tartrazine at doses up to 5000 mg/kg BW. This finding is consistent with that of the present study, which also revealed a lack of acute toxicity at doses up to 5000 mg/kg BW. This finding expands the safe range of food colors investigated in this study.

The LD_50_ is considered a tool for estimating the acute toxicity of any substance, and it has great significance in predicting the risks of any chemical, natural, or biological agent, especially in the medical and biological fields when it has been a quantal response [34]. In the present study, the LD_50_ of the commercial food color additives Sunset Yellow and Carmoisine were 6.518 g/kg BW and Tartrazine was 11.474 g/kg BW. However, the LD_50_ of the standard food dyes used in this study was 11.474 g/kg BW. According to JECFA [35], the LD_50_ values of Carmoisine and Sunset Yellow in rats were 8–10 g/kg BW, respectively, whereas the oral acute toxicity of Carmoisine in rats was 11500 mg/kg BW, which has been classified as non-toxic [36]. On the other hand, the administration of Tartrazine dye did not show any toxic effects even when a single high dose of 11.25 g/kg BW was administered orally or intraperitoneally [37]. Al-Mashhedy and Fijer [38] demonstrated that Tartrazine had LD_50_ of 4166.66 mg/kg BW. Other studies have shown that tartrazine exerts an LD_50_ of 10 g/kg BW on oral administration [13, 39, 40]. Furthermore, the results of Mannir *et al*. [41] indicated that the LD_50_ of different standard industrial color additives should exceed 5000 mg/kg BW.

The acute toxicity signs observed in morbid animals after dosing with food colors included depression, anorexia, muscular weakness, weak response to external stimuli, paralysis and inability to move, recumbence, and death. The severity of symptoms was directly proportional to the dose of the food color, and the interval between the onset of toxicity signs and death was lower for the commercial dyes than for the standard ones. This indicates that the commercial dyes contain toxic substances or impurities that contribute to the death of the dosed rats. The potential toxicity (rating) of any substance can be classified according to its LD_50_ in laboratory animals [42]. According to the Hodge and Sterner toxicity scale [43], the food dyes used in this study were considered slightly toxic. The acute toxicity analysis revealed signs of food color toxicity, and the severity of the signs was dosage dependent. In this study, more severe toxic signs and symptoms appeared in the animals when the color dosage increased. It has been observed that the route of administration is an important method for evaluating the toxicological influences of food additives [44, 45]. In this context, food color additives have toxic and carcinogenic effects when administered orally to laboratory animals [46].

To summarize, this study is innovative in many important ways, including firstly, comparative investigation. While it is well-known that commercial food dyes are frequently of inferior quality to standard dyes, FTIR spectroscopy was used in this study to provide a thorough comparative investigation of the chemical structures and functional groups of these dyes. Second, thermal properties: the current work contributes to our understanding of the physical properties of food color additives by determining the melting temperatures of both commercial and standard dyes. This method can be useful for determining whether commercial dyes are pure or have been adulterated. Third, evaluation of acute toxicity: a key contribution was the use of a range-finding study to assess the acute toxicity of these dyes in Wistar albino rats. This meticulous methodical approach offers tangible evidence of the toxicity levels of commercial versus standard dyes and aids in the establishment of the median LD_50_. Fourth, real-world relevance: this study tackles a pressing and practical issue by assessing dyes utilized locally in processed foods. The practical significance of the results is particularly beneficial for regulatory and public health organizations. Finally, fraud detection: a crucial component of the current investigation is the identification of various salt percentages in commercial dyes, which may indicate fraud. This feature draws attention to ethical and quality problems in the commercial sector. All these factors combined make our work a substantial contribution to toxicology and food safety, offering both practical implications and scientific insights.

## Conclusion

This investigation confirms differences between commercial food dyes available in local markets and imported standard dyes. They differ in terms of their chemical and toxicological aspects, indicating more impurities in commercial dyes. This is evidence of commercial fraud in the commercial colors that showed differences in the melting degree relative to the standard dyes and the appearance of clinical symptoms in the dosed animals that ended with their death, which was faster than that when pure food dyes were administered. This means that these food colors might not only affect animals but also influence consumer health. Therefore, the responsible authorities must monitor all types of dyes, either local or imported and to tighten control over their use. In addition, more studies should be conducted on food colors in terms of safety in different environments and strict control of commercial fraud operations in food production factories. The limitations of the study and future work can be shortened in several ways. First, regarding sample size and diversity, this study employed a limited number of Wistar albino rats. Future studies should employ larger and more diverse sample sizes to validate the findings. Second, duration of exposure: the study focused on acute toxicity over a short period. Thus, studies on prolonged exposure are required to fully understand the long-term effects of these dyes. Third, chemical analysis: while FTIR spectroscopy produced useful data, other analytical techniques, such as mass spectrometry, may offer more precise information regarding the chemical composition of dyes. Fourth, environmental factors: the present study did not account for environmental factors that may affect the toxicity of the dyes, such as interactions with other food ingredients or diverse storage conditions.

## Data Availability

The supplementary data can be made available by the corresponding author upon request.

## Authors’ Contributions

DAQ: Performed the experiments, studied scientific literature on the topic, and wrote the initial draft of the manuscript. IJL: Conceived the study and reviewed and revised the manuscript. Both authors have read and approved the final manuscript.
